# New β-Cyclodextrin Entrapped in Polyethyleneimine Film-Modified Electrodes for Pharmaceutical Compounds Determination

**DOI:** 10.3390/s131216312

**Published:** 2013-11-28

**Authors:** Luminţa Fritea, Mihaela Tertiş, Cecilia Cristea, Robert Săndulescu

**Affiliations:** Analytical Chemistry Department, Faculty of Pharmacy, Iuliu Haţieganu University of Medicine and Pharmacy, 4 Pasteur St., Cluj-Napoca 400349, Romania; E-Mails: Fritea.Luminita@umfcluj.ro (L.F.); mtertis@chem.ubbcluj.ro (M.T.); rsandulescu@umfcluj.ro (R.S.)

**Keywords:** β-cyclodextrin modified electrode, ascorbic acid, uric acid, square wave voltammetry, polyethyleneimine, pharmaceutical products

## Abstract

The electrochemical behavior of ascorbic acid and uric acid on glassy carbon bare electrodes and ones modified with *β*-cyclodextrin entrapped in polyethyleneimine film has been investigated using square wave voltammetry. The electrode modification was achieved in order to separate the voltammetric peaks of ascorbic acid and uric acid when present in the same solution. On the modified electrodes the potential of the oxidation peak of the ascorbic acid was shifted to more negative values by over 0.3 V, while in the case of uric acid, the negative potential shift was about 0.15 V compared to the bare glassy carbon electrode. When the two compounds were found together in the solution, on the bare electrode only a single broad signal was observed, while on the modified electrode the peak potentials of these two compounds were separated by 0.4 V. When the uric acid concentration remained constant, the peak intensity of the ascorbic acid is increased linearly with the concentration (r^2^ = 0.996) and when the ascorbic acid concentration remains constant, the peak intensity of the uric acid increased linearly with the concentration (r^2^ = 0.992). FTIR measurements supported the formation of inclusion complexes. In order to characterize the modification of the electrodes microscopic studies were performed. The modified electrodes were successfully employed for the determination of ascorbic acid in pharmaceutical formulations with a detection limit of 0.22 μM.

## 1. Introduction

Ascorbic acid (AA), with its tremendous antioxidant effect, is an important component of the human diet. It plays an important role in bioelectrochemistry, neurochemistry and clinical diagnostics applications [1]. Uric acid (UA) is the primary end product of purine metabolism. Abnormal concentrations of UA indicate diseases such as gout, hyperuricaemia and Lesch-Nyhan syndrome. Colorimetric, enzymatic and electrochemical methods have been used for the detection and quantification of these two substances [2]. Electrochemical methods based on the use of bare electrodes are not very selective. In the case of ascorbic and uric acid the major problem of the electrochemical method is the interference between them taking into account that their oxidation potential values are very close to each other [3]. In order to overcome this problem various modified electrodes were developed for simultaneous selective determination of the two species with and without other interferences [1–17].

For this purpose many sensors based on modified glassy carbon electrodes (GCE) involving different complex methods for their modification are reported in the literature. Among these, a sensor was developed through electrodepositing gold nanoparticles on a GCE modified with a film of polyimidazole for the simultaneous determination of ascorbic acid, dopamine, uric acid and tryptophan with a limit of detection (LOD) of 2 μM for AA [6]. Other authors used a poly(3-(5-chloro-2-hydroxy-phenylazo)-4,5-dihydroxynaphthalene-2,7-disulfonic acid) film to modify GCE in order to simultaneously detect ascorbic acid, dopamine and uric acid, obtaining a LOD of 1.43 μM for AA [8]. An electropolymerized film of 4-aminobutyric acid was prepared on the surface of a GCE, sensor which was used for the determination of ascorbic acid, dopamine and uric acid in mixture with a LOD of 5 μM for AA [10].

In many studies cyclodextrins (CDs) were included in the composition of the electrode modifier, enhancing the sensitivity and selectivity of the AA and UA determinations [18–21]. Cyclodextrins are macrocyclic oligosugars consisting of 6, 7 or 8 glucopyranose units linked by α-1−4 bonds. CDs can bind a variety of guest molecules inside their torus-shaped cavities and serve as a model of host site [22]. CDs with their large hydrophobic cavities of variable size and numerous ways of chemical modification are the subject of intensive electrochemical research including both their behavior in homogeneous solutions [23] and in thin films attached to the electrode surfaces [24]. The CDs based sensors were used with good results for the determination of *p*-nitrophenol [25,26], glucose [27], α-fetoprotein [28], dopamine [29,30], cathecol and xanthine [31,32], serotonine and dopamine [33], quercetin and hydrogen peroxide [34]. In the literature are reported some sensors having β-CD in their composition that were used for the determination of: AA in presence of UA [21], cathecol [20], UA and dopamine [19].

Polyethyleneimine (PEI) is a cationic polymer used for the entrapment of several molecules in various biosensors [35]. When used as an entrapment polymer, PEI offers the advantage of retaining the biomolecule at the surface of the electrode without stressing it with a supplementary electropolymerization process [36,37]. Another advantage of PEI consists in its partial solubility in water, property that is used in the development of biosensors.

In this study, we report a simple method for the modification of GCE with the polymeric films of PEI with β-cyclodextrin (β-CD) for the analysis of ascorbic and uric acids, separately and together in the same solution. FTIR spectra explained the electrochemical response.

These modified electrodes were applied successfully for the dosage of ascorbic acid from pharmaceutical products, the possible interferences with the excipients being also tested. The presence of the β-CD in the polymeric film allowed to better separation of the AA and UA peaks, increased their signals and shifted their oxidation potentials to more negative values, highlighted the advantages of this modified electrode compared with the bare one.

This novel sensor based on glassy carbon electrode modified with β-CD entrapped in PEI film has many advantages compared with other sensors used for AA detection. Among them, a simple, fast method for the modification of the electrode, fast and simple analysis, and good values for the analytical parameters can be mentioned:. To our knowledge this is the first sensor with β-CD used for the determination of AA with such a low LOD.

## 2. Experimental Section

### 2.1. Materials and Methods

β-CD was purchased from Merck (Whitehouse Station, NJ, USA), UA, AA, sodium dihydrogen phosphate and sodium monohydrogen phosphate were purchased from Sigma Aldrich (St. Louis, MO, USA). Phosphate buffer solution (PBS, 0.1 M, pH 7.2) was used. All the reagents were used without further purification and they were all of analytical grade. All aqueous solutions were prepared with ultrapure water. All the measurements were performed at room temperature.

The electrochemical experiments were carried out with an Autolab PGSTAT 12 potentiostat (EcoChemie, Utrecht, Netherlands) equipped with GPES 4.9 software, using a traditional three-electrode system. GCE purchased from Bioanalytical Systems (BAS, West Lafayette, IN, USA) was used as the working electrode, with Ag/AgCl as reference electrode and a platinum wire as the counter electrode. The FTIR spectra were measured in a Jasco FT/IR-4100 spectrophotometer (Jasco, Tokyo, Japan) equipped with Jasco Spectra Manager Version 2 software on wave number 550–4,000 cm^−1^. The solid samples were obtained after evaporation of the same solvent used in the electrochemical analysis.

Square wave voltammetry (SWV) employed a frequency of 15 Hz and amplitude of 25 mV. The mixtures were obtained by vortexing the solutions for 3 min at 400 rpm. The UA solution was prepared using ultrasonication during 10 min and heating up to 60 °C to solve the powder. For the real sample analysis the square wave voltammograms were obtained by scanning potential from −0.3 to 1.0 V with frequency of 25 Hz and amplitude of 25 mV.

The spectrophotometry determinations were performed on SPECORD 250 PLUS spectrophotometer (Analitik Jena, Jena, Germany) equipped with WinAspect software. Ascorbic acid was determined by spectrophotometry in UV domain at 266 nm. The microscopic characterization of the modified electrodes surface was obtained using a WiTec Alpha 300R confocal Raman microscope (WiTec, Knoxville, TN, USA).

### 2.2. Preparation of the Working Electrodes

Solutions of PEI (5 mg/mL) and PEI (1 mg/mL) were prepared by dissolving the necessary weight of PEI in the solvent composed of water: alcohol 1:1. The solution of PEI (5 mg/mL) + β-CD (1%) was obtained by dissolving the necessary amount of β-CD in the solvent in which then PEI was dissolved. The other solution of PEI (1 mg/mL) + β-CD (0.2%) was prepared by diluting 5 times the above solution. Five types of working electrodes were used: bare GCE, GCE modified with PEI (5 mg/mL), PEI (1 mg/mL), GCE modified with PEI (5 mg/mL) + β-CD (1%), PEI (1 mg/mL) + β-CD (0.2%). 6 μL of the PEI solution, simple and modified with β-CD, was dropped on the electrode and then was dried for 30 min at room temperature.

### 2.3. Procedure for Real Samples Analysis

The injectable solutions from ten vials of Vitamina C^®^ (Arena, Bucharest, Romania) were mixed together for homogenization and then were used to obtain 10^−2^ M considering the declared concentration, a stock solution adding an appropriate volume of PBS (pH 7.2, 0.1 M). In the case of ascorbic acid oral solution from the second analyzed pharmaceutical formulation, Vitamina C^®^ (Biofarm, Bucharest, Romania), five bottles were mixed together following the same procedure mentioned above for the injection solution. The stock solutions were prepared daily and maintained in the dark, protected from light and at constant temperature. In order to quantify the ascorbic acid concentration from the pharmaceutical samples, the analytical data obtained from the calibration curves were used.

## 3. Results and Discussion

### 3.1. Electrochemical and FT-IR Analysis on PEI+β-CD Glassy Carbon Modified Electrodes

#### 3.1.1. Square Wave Voltammetry of Uric and Ascorbic Acid on Glassy Carbon Electrode Modified with Polymeric Film of PEI + β-CD and FT-IR Spectra of the Complexes

**Figure 1.**
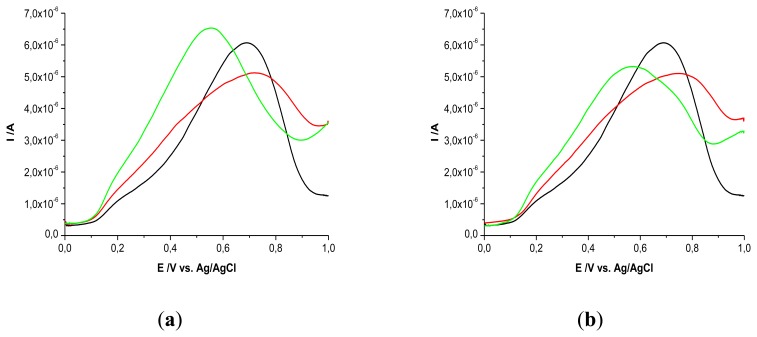
SWVs of UA 10^−3^ M in PBS (0.1 M; pH 7.2) on bare GCE (black); GCE+PEI (red); GCE + PEI + CD (green) (**a**) PEI concentration: 5 mg/mL, β-CD concentration: 1%; (**b**) PEI concentration: 1 mg/mL, β-CD concentration: 0.2%.

The two analytes (UA and AA) were tested on GCEs modified with PEI + β-CD films using SWV. There were noticeable differences in peak potential and peak height on GCEs modified with PEI + β-CD film in comparison with bare GCEs and PEI/GCEs. UA was detected on bare GCE at 0.684V *vs* Ag/AgCl, the peak potential was shifted with 0.150 V *vs* Ag/AgCl on PEI (5 mg/mL) + β-CD (1%)/GCE, Figure 1a, and with 0.144V on PEI (1 mg/mL) + β-CD (0.2%)/GCE, Figure 1b, due to the complexation between β-CD from the film and the UA from solution.

Ramirez Berriozabal *et al.* [3] obtained a positive shift for the oxidation peak of the uric acid on β-CD/carbon paste electrode CPE. The guest molecule of UA is entrapped in the cavity of the CD making it easier to be oxidized because it is retained on the film at the surface of the electrode. As it can be observed from Table 1, the peak height of UA on bare GCE was bigger than the one that obtained on the modified GCEs. In the case of UA, even the PEI (5 mg/mL) + β-CD (1%)/GCE did not improve the current response, Table 1.

**Figure 2.**
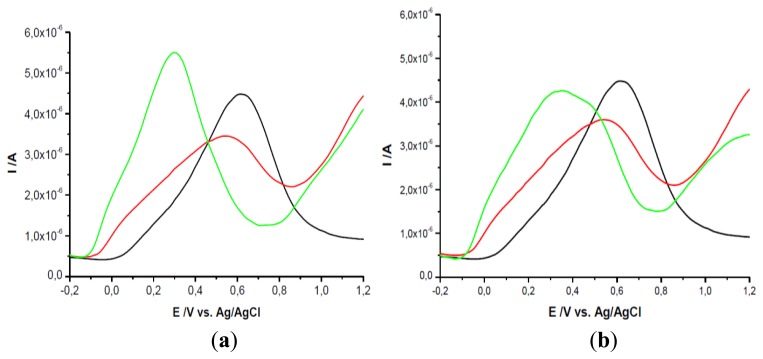
SWVs of AA 10^−3^ M in PBS (0.1 M; pH 7.2) on bare GCE (black); GCE + PEI (red); GCE + PEI + CD (green) (**a**) PEI concentration: 5 mg/mL, β-CD concentration: 1%; (**b**) PEI concentration: 1 mg/mL, *β*-CD concentration: 0.2%.

AA presented an oxidation peak at 0.611 V *vs.* Ag/AgCl on bare GCE with an intensity current of 3.81 μA. On the modified GCEs the peak potential was shifted to more negative values, the most remarkable shift being noticed on PEI (5 mg/mL)+β-CD (1%)/GCE with a value of 0.316 V *vs.* Ag/AgCl, Figure 2.

In the case of AA oxidation peak, Ramirez Berriozabal *et al.* have reported a negative shift of 0.260 V using *β*-CD/CPE; Colleran and Breslin obtained a cathodic shift of 0.080–0.135 V on PEDOT/S-*β*-CD/Au [3,20]. The current intensity was grown only on PEI (5 mg/mL) + β-CD (1%)/GCE with 0.82 A compared to bare GCE, Table 2.

This behavior can be explained by the inclusion complexes formed between AA and β-CD, complexation which was more effective for AA than for UA. The peak area varied in a similar way with peak height in both cases.

The increase of the current intensity can be attributed to the preconcentration of the analyte at the electrode surface due to the complexation with cyclodextrin which retains more molecules at the surface of the electrode. The negative shift of peak potential can be explained by the mediator effect of cyclodextrin: its cavity acts like a tunnel which directs the electrons to the electrode surface for the redox process.

In order to understand the electrochemical behavior of the two analytes on the GCEs modified with PEI + β-CD, spectral studies were performed: the complexes were obtained in PB at pH 7.2 and then the solvent was evaporated and solid samples were used for the measurements.

**Figure 3.**
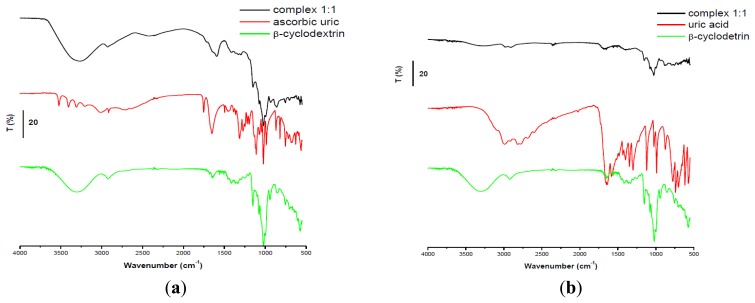
FTIR spectra of β-CD, AA (**a**); UA (**b**) and inclusion complexes.

FTIR spectra of the complexes between AA/UA and β-cyclodextrin are shown in Figure 3. Analyzing the spectra of the two complexes it can be observed that some specific bands of cyclodextrin were attenuated and shifted as follows: 3,313 cm^−1^ (O–H stretch, H–bonded), 2,924 cm^−1^ (C–H stretch in alkanes), 1,338–1,020 cm^−1^ (C–O stretch in alcohols and ethers), 946–754 cm^−1^ (carbohydrates bends), 605–574 cm^−1^ (ether bend).

Two AA bands at 1,652 cm^−1^ (C=C stretch) and 683 cm^−1^ (=C–H bend) were diminished and shifted in the spectra of the complex while many bands disappeared after complexation: 3,523, 3,406, 3,310, 3,206 cm^−1^ (four O–H stretches are hidden in the broad band at 3,277 cm^−1^ from β-CD), 3,005 cm^−1^ (=C–H stretch), 1,752 cm^−1^ (C=O stretch in carbonyl), 1,496 cm^−1^ (C–C stretch in–ring), 1,455 cm^−1^ (C–H bend), 1,387 cm^−1^ (C–H rock in alkanes) 1,312–1,110 cm^−1^ (C–O stretch), 987–683 cm^−1^ (=C–H bend) (Figure 3a).

Some bands of UA were diminished and shifted in the spectra of the complex: 2,986 cm^−1^, 2,916 cm^−1^ (CH_3_ asymmetric and N-H stretch in imidazole), 1,024 cm^−1^ (N-H stretching, C-N stretch in pyrimidine), 874 cm^−1^ (N-H out-of-plane bending). Other bands of the analyte have disappeared: 2,817–2,024 cm^−1^ (ν CH_3_ asymmetric and N-H stretch, asymmetric stretch of C=C), 1,652–1,435 cm^−1^ (C=O stretch in unsaturated carbonyl, asymmetric deformation of NH_3_, CH_2_–CO bending, δ CH_2_ scissoring), 1,398–1,348–1,299 cm^−1^ (CH_2_-CO deformation, ν C=O, ν CN in imidazole), 1,120 cm^−1^ (N-H stretching, C-N stretch in pyrimidine), 989–572 cm^−1^ (C–C stretch, N–H out-of-plane bending, C–N stretching of aromatic) (Figure 3b). It can be observed the formation of inclusion complexes between UA and β-CD, AA and β-CD, respectively.

#### 3.1.2. Square Wave Voltammetry of Ascorbic and Uric Acid in Mixture on PEI + β-CD/GCEs and FT-IR Spectra of the Complexes

##### (a) At Constant Concentration of UA (10^−3^ M)

**Figure 4.**
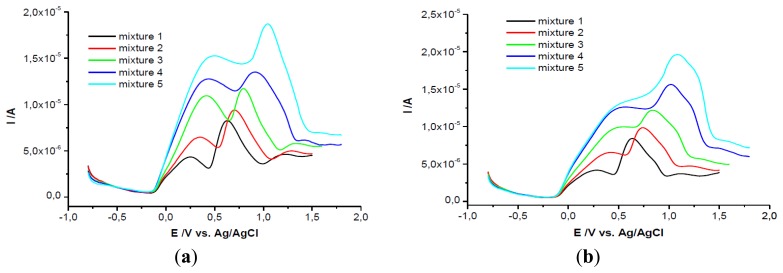

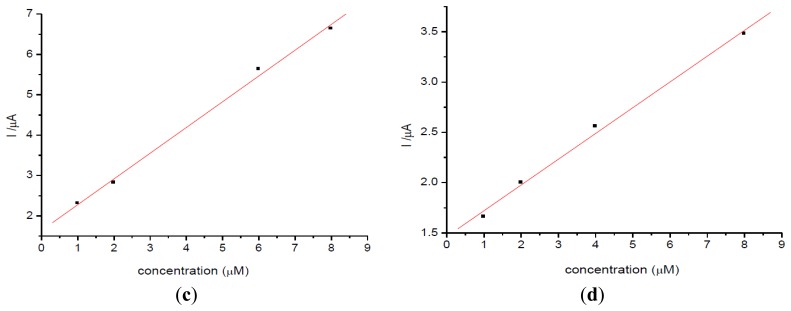
SWVs of the five mixtures on GCE/PEI (5 mg/mL) + *β*-CD (1%) (**a**) and on GCE/PEI (1 mg/mL) + β-CD (0.2%) (**b**) (mixture 1) 10^−3^ M both substances, (mixture 2) 2 × 10^−3^ M AA, (mixture 3) 4 × 10^−3^ M AA, (mixture 4) 6 × 10^−3^ M AA, (mixture 5) 8 × 10^−3^ M AA acid, all mixtures contained 10^−3^ M UA; (**c**) Calibration curve for (a); (**d**) Calibration curve for (b).

It is very well known the interference between ascorbic and uric acid in electrochemical analysis studies. In order to separate the electrochemical signal of the two compounds when they are in mixture in the same solution, GCEs modified with PEI + β-CD films in two concentrations were used. These modified electrodes were tested in solutions of a constant concentration in UA and at different concentrations of AA (Figure 4).

Then the results were compared with those obtained on bare GCE and on PEI/GCE. The voltammograms show two peaks for these two substances only when the PEI + β-CD/GCEs were used, note that on bare GCE, on PEI/GCE and on β-CD/CPE only one peak is observed, (data not shown).

The peak separation on the GCEs modified with PEI (5 mg/mL) + β-CD (1%) films was greater than 0.42 V (for mixture 1 was 0.422 V and grew up until 0.695 V for mixture 5). The second peak attributed to UA (the same concentration in all mixtures) remained almost constant (relative deviation for mixtures 2–5 was ±2.38%). The first peak, corresponding to AA oxidation, grew linearly with the concentration in AA (Ip(μA) = 1.637 + 638.167 C_AA_(mM); r^2^ = 0.996) (Figure 4c).

Even though the voltammograms recorded on PEI (1 mg/mL) + β-CD (0.2%)/GCE revealed a peak potential separation of more than 0.43 V (for mixture 2 the difference was 0.432 V and it reaches 0.742 V in mixture 5), the AA peak height was not as large as on the PEI (5 mg/mL) + β-CD (1%)/GCE, the slope of the calibration line being 2.5 times smaller. The UA peak remained almost constant only in the first three mixtures and then it increased. This behavior may be interpreted in the following manner: if the CD concentration is low and the AA concentration is increased, AA is forming more readily the complex with CD than the UA, then increasing concentrations of UA remain in solution and the UA, in its uncomplexed form is detected at the electrode. The AA peak height grew linear when its concentration was increased (Ip(μA) = 1.464 + 256.173 C_AA_ (mM); r^2^ = 0.994) (Figure 4d).

Other authors managed to discriminate between the voltametric signals of AA and UA, too, using different sensors, like: *β*-CD incorporated in a carbon paste electrode (ΔE = 0.376 V) [3]; gold nanoparticles-β-cyclodextrin-graphene-modified electrode (ΔE = 0.324 V) [19] and β-cyclodextrin modified copolymer of sulfanilic acid and N-acetylaniline on glassy carbon electrode (ΔE = 0.275 V) [21], most of them needed a laborious method of modification.

**Figure 5.**
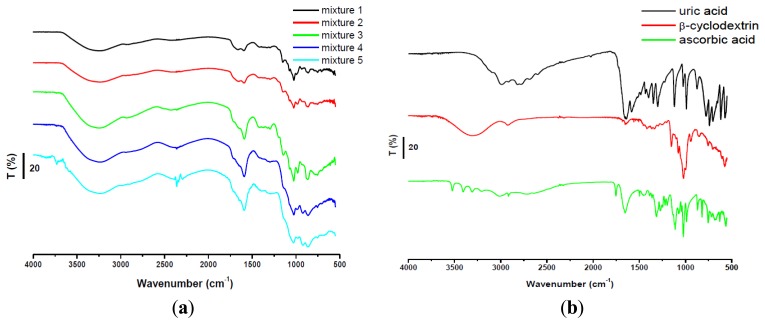
(**a**) FTIR spectra for the five mixtures: (mixture 1) β-CD: UA: AA = 1:1:1, (mixture 2) β-CD: UA: AA = 1:1:2, (mixture 3) β-CD: UA: AA = 1:1:4, (mixture 4) β-CD: UA: AA = 1:1:6, (mixture 5) β-CD: UA: AA = 1:1:8; (**b**) FTIR spectra for UA, β-CD and AA.

FTIR measurements were used to explain the formation of inclusion complexes that could influence the electrochemical behavior of these compounds. As it can be observed from FTIR spectra, the growing concentration of AA determined the growth of the band from 1,590 cm^−1^ which was the shift of the peak of the UA from 1,580 cm^−1^ (asymmetric deformation of NH_3_) (Figure 5b). A possible explanation would be the fact that AA is preferentially allowed to enter the cavity of the CD leading to an increasing accumulation of uncomplexed UA, Figure 5a.

##### (b) At Constant Concentration of AA (10^−3^ M)

**Figure 6.**
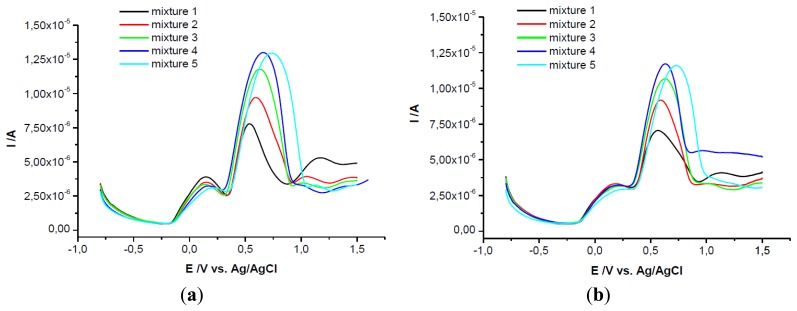

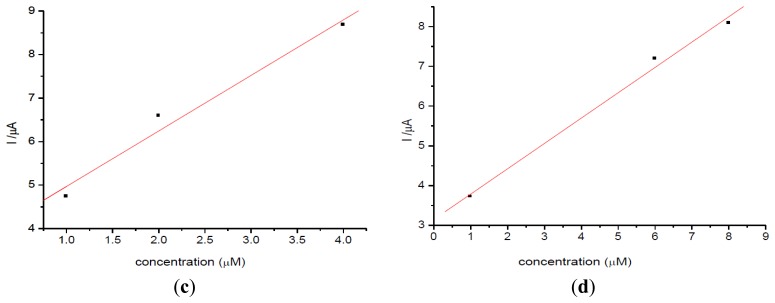
SWVs of the five mixtures on GCE/PEI (5 mg/mL) + β-CD (1%) (**a**) and on GCE/PEI (1 mg/mL) + β-CD (0.2%) (**b**) (mixture 1) 10^−3^ M both substances, (mixture 2) 2 × 10^−3^ M UA, (mixture 3) 4 × 10^−3^ M UA, (mixture 4) 6 × 10^−3^ M UA, (mixture 5) 8 × 10^−3^ M UA, all mixtures contained 10^−3^ M AA; (**c**) calibration curve for (a); (**d**) calibration curve for (b).

When the mixtures were analyzed on PEI (5 mg/mL) + β-CD (1%)/GCE the peak potential separation was 0.411 V for the first mixture, reaching 0.575 V for the fifth mixture. The current intensity of UA grew linearly for the first three mixtures (Ip(μA) = 3.695 + 1275 C_UA_ (mM); r^2^ = 0.976) (Figure 6a,c).

On the PEI (1 mg/mL) + β-CD (0.2%)/GCE the difference in peak potential was 0.457 mV for the second mixture and 0.611 mV for the last one. The increasing current intensity of UA was linear having the following equation: Ip(μA) = 3.146 + 638.076 C_UA_ (mM); r^2^ = 0.992 (Figure 6b,d).

**Figure 7.**
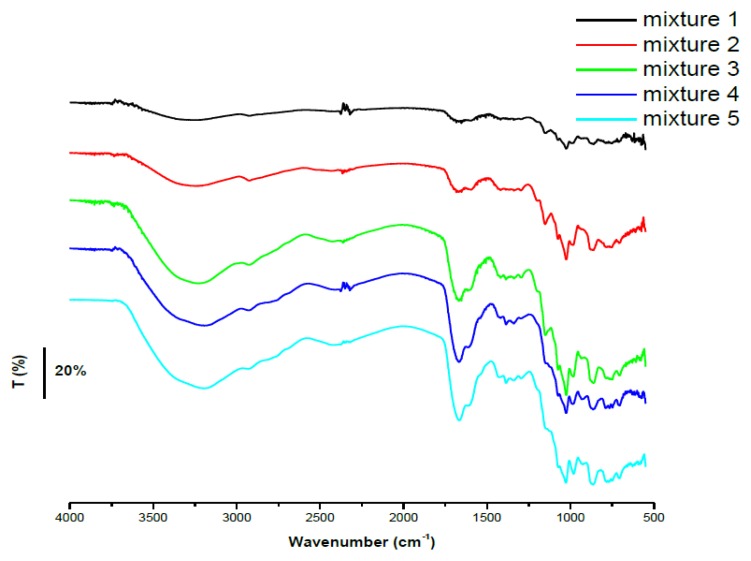
FTIR spectra for the five mixtures: (mixture 1) β-CD: AA: UA = 1:1:1, (mixture 2) β-CD: AA: UA = 1:1:2, (mixture 3) β-CD: AA: UA = 1:1:4, (mixture 4) β-CD: AA: UA = 1:1:6, (mixture 5) β-CD: AA: UA = 1:1:8.

The FTIR spectra for the five mixtures showed two bands that were increasing gradually with the concentration of UA. The uric acid peaks from 1,580 cm^−1^ (asymmetric deformation of NH_3_) and 1,640 cm^−1^ (C=O stretch) were shifted in the case of the complexes and appeared at 1,600 cm^−1^ and 1,660 cm^−1^, Figure 7.

### 3.2. Microscopic Images of the Modified Electrode Surface

**Figure 8.**
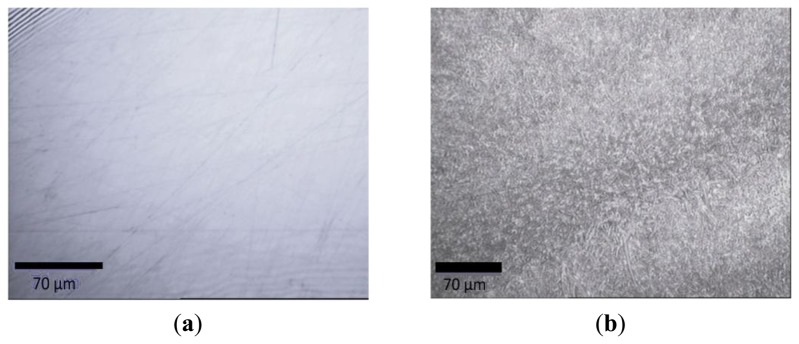
Microscopic images for: (**a**) PEI (5 mg/mL)/GCE and (**b**) PEI (5 mg/mL) + CD (1%)/GCE.

The electrodes modified with thin films of PEI (5 mg/mL) and PEI (5 mg/mL) + CD(1%) on GCE were characterised using a confocal Raman microscope and the obtained images are presented in Figure 8a,b.

Comparing the two images it can be clearly observed the difference between the two types of modified electrodes due to the presence of β-CD. In the case of the electrode modified with PEI (5 mg/mL) + CD(1%), the β-CD structures were uniformly and homogenously distributed in the PEI film covering the entire electrode surface, Figure 8b.

### 3.3. Pharmaceutical Samples Analysis

#### 3.3.1. Electrochemical Determination of the Ascorbic Acid in Pharmaceutical Products and Interference Studies

The PEI + *β*-CD modified electrodes were applied for the assay of ascorbic acid in two commercial drug formulations: oral solution Vitamina C^®^ Biofarm (dietary supplement) and injectable solution Vitamina C^®^ Arena.

One vial of 5 mL ascorbic acid injectable solution from Arena Group SA^®^ contains, besides 750 mg vitamin C, other excipients: NaOH, 3.75 mg sodium metabisulphite and water for injections. Therefore, a study of electrochemical interference from sodium metabisulphite, which is known as a common reducing agent was initiated with 215.26 μM solution of ascorbic acid with 116 μM solution of sodium metabisulphite (in the same ratio as in Vitamina C^®^ Arena). Measurements of the peak currents for each solution were repeated three times and the average current values were used. If the average signal for ascorbic acid in the presence of the interferent, in comparison with the one for ascorbic acid alone, was altered by less than ±5%, we considered that the interference was negligible, in agreement with literature [38,39].

**Figure 9.**
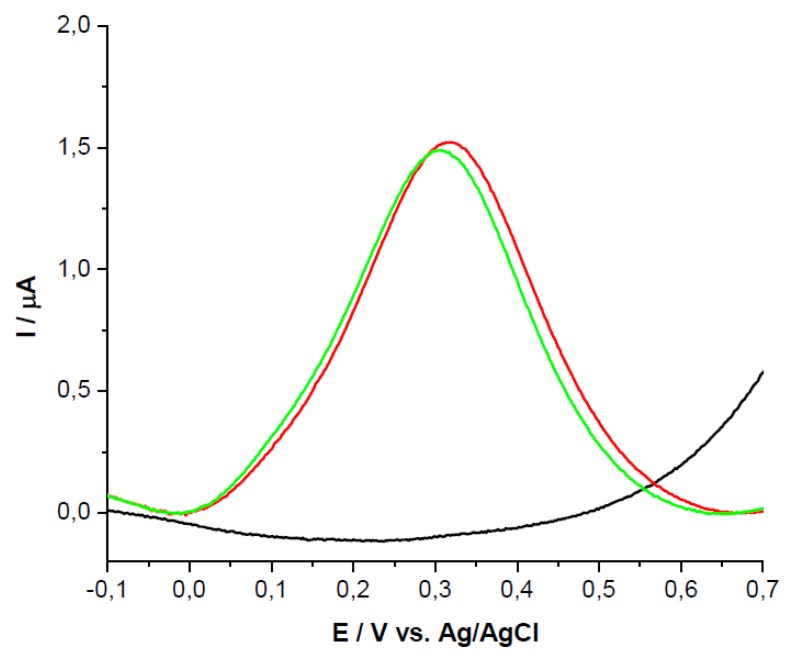
SWVs for: 215.26 μM AA solutions in the absence (red) and in the presence of 116 μM sodium metabisulphite (green) compared with the electrolyte (0.1 M PB; pH 7.2) (black).

The voltammetric signals for AA recorded on the PEI + *β*-CD modified electrode, in the presence and in the absence of the sodium metabisulphite are presented in Figure 9. The sodium metabisulphite slightly influenced the ascorbic acid signal, decreasing it with 2.61%.

The obtained data allowed us to conclude that these compounds did not interfere or at least the interference was negligible. This is the reason why the calibration curve was obtained using standard ascorbic acid without sodium metabisulphite.

**Figure 10.**
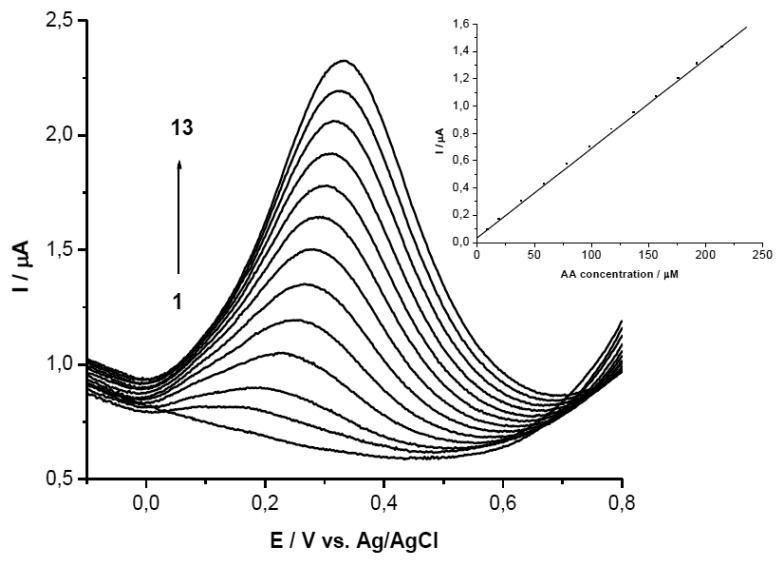
SWVs for AA solution with different concentrations: (1) 0 μM; (2) 9.99 μM; (3) 19.96 μM; (4) 39.84 μM; (5) 59.64 μM; (6) 79.36 μM; (7) 99 μM; (8) 118.5 μM; (9) 138.06 μM; (10) 157.48 μM; (11) 176.81 μM; (12) 193.07 μM; (13) 215.26 μM (Inset: calibration curve).

The calibration curve was determined by adding equal aliquots of ascorbic acid pro analysis (2 × 5 μL and 10 × 10 μL). The equation for the calibration curve was: Ip(μA) = 0.035 + 0.007 × 10^−5^ C_AA_(μM); r^2^ = 0.999 (Figure 10).

The recovery rates of the active substance in Vitamina C^®^ Arena containing ascorbic acid were between 98.25% and 101.02%, interferences with all other formulation excipients being negligible, Table 3.

The method specified in Romanian Pharmacopeia for the dosage of ascorbic acid from the injectable solution is polarimetry. The ascorbic acid concentration calculated with the polarimetric method gave a result of 95.3% compared with the quantity declared by the manufacturer, in agreement with the imposed limits (±5% deviation) [40].

**Figure 11.**
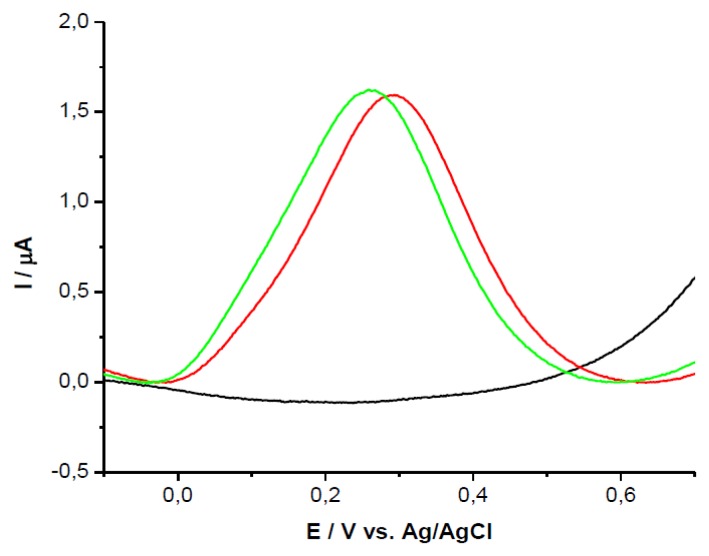
SWVs for: 215.26 μM AA solutions in the absence (red) and in the presence of 215.26 μM propylene glycol (green) compared with the electrolyte (0.1 M PB; pH 7.2) (black).

One vial of 10 mL ascorbic acid oral solution from Biofarm^®^ contains 1,000 mg vitamin C dissolved in 10 mL propylene glycol and water. For the determinations of AA from Vitamina C^®^ Biofarm there was used another calibration curve obtained in the presence of propylene glycol because this cosolvent influenced the oxidation peak of AA shifting it to a more negative potential with almost 0.04 V and increased the peak intensity with 1.87%, Figure 11.

**Figure 12.**
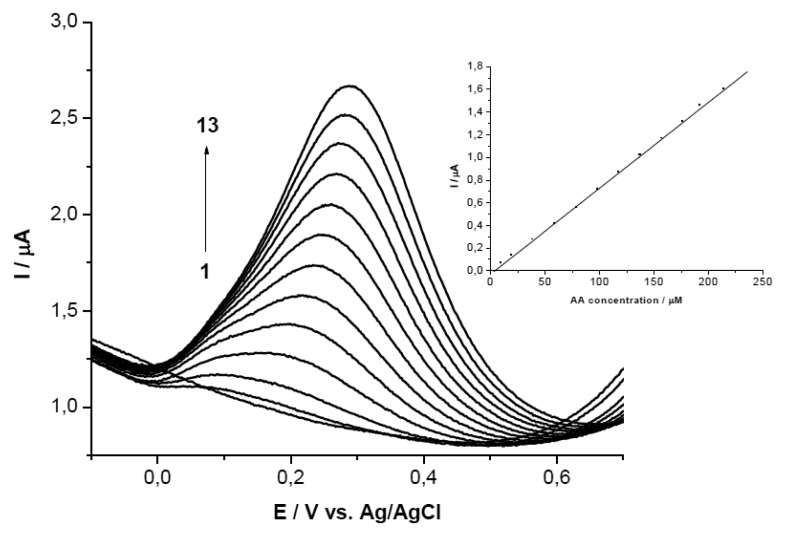
SWVs for AA solution with different concentrations: (1) 0 μM; (2) 9.99 μM; (3) 19.96 μM; (4) 39.84 μM; (5) 59.64 μM; (6) 79.36 μM; (7) 99 μM; (8) 118.5 μM; (9) 138.06 μM; (10) 157.48 μM; (11) 176.81 μM; (12) 193.07 μM; (13) 215.26 μM; in the presence of propylene glycol (Inset: calibration curve).

The equation for the calibration curve was: Ip(μA) = −0.031 + 0.008 10^−5^ C_AA_(μM); r^2^ = 0.999 (Figure 12).

There were found recovery rates between 97.29% and 99.06% in good accordance with the Romanian Pharmacopeia specification (±5% deviation) (Table 3) [40].

The present developed sensor was used to calculate the detection limit for AA using SWV over the concentration rage of 9.99–215.26 μM. The detection limit (S/N = 3) was calculated to be 0.22 μM, value which is comparable with the lowest LOD reported in literature, being 50 times lower than other methods employing electrodes modified with CDs, Table 4 [1,6–21].

#### 3.3.2. UV-Vis Determination

In order to compare the electroanalytical results, ascorbic acid solutions (standard and obtained with the investigated pharmaceutical Vitamina C^®^ Arena) were analyzed by spectrophotometry at 266 nm (the characteristic AA wavelength). A calibration curve was drawing in the 1 μM−100 μM range, with the following equation: A = 0.151 + 0.145 C_AA_(μM); (r^2^ = 0.994; RSD = 2.22%). The recovery rates for the pharmaceutical samples were between 100.23% and 103.89%, RSD = 3.21%, results which were in good agreement with those obtained by the electrochemical method. The UV-Vis method for the determination of the ascorbic acid in solution is the standard method recommended also by European Pharmacopoeia and used by the pharmaceutical companies [41].

## 4. Conclusions

The electrochemical behavior of ascorbic acid and uric acid was investigated on glassy carbon electrode, bare and modified with β-cyclodextrin in polyethyleneimine film using square wave voltammetry. The electrode modifications by β-cyclodextrin allowed the separation of the voltammetric signals and the shifting to more negative of the oxidation peaks for the two compounds. At constant concentration of the uric acid, the intensity of the oxidation peak for the ascorbic acid increased linearly with the concentration. When the concentration of the ascorbic acid was kept constant, the intensity of the oxidation peak for the uric acid increased linearly with the concentration.

FTIR measurements explained the electrochemical behavior of ascorbic acid and uric acid in solution. The increasing concentration of ascorbic acid determined the growth of the band which corresponds to the asymmetric deformation of NH_3_ in uric acid. This behavior is probable due to the fact that ascorbic acid was the first incorporated into β-CD, forming an inclusion complex, while the uric acid concentration increased in solution. Apparently AA is preferentially allowed to enter the cavity of the CD.

The PEI + β-CD/GC modified electrodes allowed simultaneous determination of ascorbic acid and uric acid due to the well separation of signals corresponding to the electrochemical oxidation processes of these compounds.

The modified electrodes applied for the detection and dosage of ascorbic acid from two pharmaceutical products presented reliable performance: low detection limit, good linear range, the absence of interferences in the case of injectable solution. The pharmaceutical samples analysis showed good recoveries using electrochemical methods and the results were confirmed using spectrophotometric methods.

## Figures and Tables

**Table 1.** SWV parameters for 10^−3^ M UA on different electrodes.

**Electrode Type**	**Peak Potential (V)**	**Peak Height (μA)**	**Peak Area (μC)**
GCE	0.684	5.11	2.03
PEI(1 mg/mL)/GCE	0.674	2.42	1.24
PEI(5 mg/mL)/GCE	0.661	2.62	1.35
PEI(1 mg/mL) + β-CD(0.2%)/GCE	0.54	3.46	1.52
PEI(5 mg/mL) + β-CD(1%)/GCE	0.534	4.64	1.89

**Table 2.** SWV parameters for 10^−3^ M AA on different electrodes.

**Electrode Type**	**Peak Potential (V)**	**Peakheight (μA)**	**Peakarea (μ)**
GCE	0.611	3.81	1.76
PEI(1 mg/mL)/GCE	0.513	2.06	1.09
PEI(5 mg/mL)/GCE	0.505	1.85	0.99
PEI(1 mg/mL) + βCD(0.2%)/GCE	0.321	3.29	1.65
PEI(5 mg/mL) + β-CD(1%)/GCE	0.295	4.63	1.78

**Table 3.** Ascorbic acid determination in pharmaceutical products on PEI + *β*-CD/GCE by using SWV.

**Pharmaceutical Product**	**Theoretical Concentration (μM)**	**Found Concentration (μM)**	**Recovery (%)**	**RSD (%)**
Vitamina C^®^ Arena	157.48	154.72	98.25	
	176.81	175.45	99.23	1.41
	193.07	195.04	101.02	

Vitamina C^®^ Biofarm	157.48	156.01	99.06	
	176.81	173.20	97.29	1.02
	193.07	191.14	99.00	

**Table 4.** Comparison of sensitivity of the developed sensing device with previously reported sensors.

**Analyte**	**Electrode/Method**	**LOD (for AA) (μM)**	**Reference**
AA, UA, DA, Tryptophan	GNP/PImox/GCE ^a^; DPV	2.00	[6]
AA, UA	GNP/LC/GCE ^b^; DPV	-	[7]
AA, UA, DA	CDDA/GCE ^c^; DPV	1.43	[8]
AA, UA, Epinefrine	PCAC/GCE ^d^	0.40	[9]
AA, UA, DA	P-4-ABA/GCE ^e^; DPV	5.00	[10]
AA, UA, DA	GRA/Pt/GCE ^f^; CV, DPV	-	[11]
AA, UA, DA	NG/GCE ^g^; CV, DPV	2.20	[12]
AA, DA, UA	GRA/SPE ^h^; CV, DPV	0.95	[13]
AA, UA	GA/TC-GNP/Au ^i^; CV	-	[14]
AA, UA, DA	PDDA-HCNTs/GCE ^j^; DPV	0.12	[1]
AA, UA, DA	PS(III)/GCE ^l^; DPV	0.17	[15]
AA, UA	OC/GCE ^m^; CV, amperometry	10	[17]
AA, UA, DA	AuNPs–*β-*CD–Gra/GCE ^n^; SWV	10	[19]
AA, DA	(PEDOT/S-*β*-CD) films/Au ^o^; CV, Amperometry, Hydrodynamic voltammetry	-	[20]
AA, UA	*β-*CD+p-ASA+SPNAANI/GCE ^p^	-	[21]
AA, UA	*β-*CD/PEI/GCE; SWV	0.22	This work

a GNP/PImox/GCE = gold nanoparticles/overoxidized-polyimidazole composite modified glassy carbon electrode;

b GNP/LC/GCE = gold nanoparticles and l-cysteine on glassy carbon electrode;

c CDDA/GCE = poly (3-(5-chloro-2-hydroxyphenylazo)-4,5-dihydroxynaphthalene-2,7-disulfonic acid) film modified glassy carbon electrode;

d PCAC/GCE = poly(3,3_-bis[N,N-bis(carboxymethyl)aminomethyl]-*o*-cresolsulfonephthalein) modified glassy carbon electrode;

e P-4-ABA/GCE = poly(4-aminobutyric acid) modified glassy carbon electrode;

f GRA/Pt/ GCE = graphene/Pt-modified glassy carbon electrode;

g NG/GCE = Nitrogen doped graphene /GCE;

h GRA/ SPE = screen-printed electrode using an ink containing grapheme;

i GA/TC-GNP/Au = guanine/thiocytosine-gold nanoparticles/Au;

j PDDA-HCNTs/GCE = poly(diallyl dimethylammonium chloride functionalised helical carbon nanotubes on glassy carbon electrodes;

l PS(III)/GCE = poly(sulfonazo III) modified glassy carbon electrode;

m OC/GCE = organoclay film modified glassy carbon electrodes;

n AuNPs–*β-*CD–Gra/GCE = Graphene decorated with gold nanoparticles and β-CD on glassy carbon electrode;

o (PEDOT/S-*β*-CD) films/Au = Poly (3,4-ethylene dioxythiophene)/sulphated *β*-cyclodextrin Au films, deposited onto gold working electrodes;

p CD + p-ASA+SPNAANI/GCE = *β* -cyclodextrin modified copolymer membrane of sulfanilic acid and *N*-acetylaniline on glassy carbon electrode; AA = ascorbic acid; UA = uric acid; DA = dopamine; CV = cyclic voltammetry; SWV = square wave voltammetry; DPV = differential pulse voltammetry.
